# Supplementary material for: Personalized Machine Learning-Based Prediction of Wellbeing and Empathy in Healthcare Professionals
Source: Sensors (Basel). 2024 Apr 20;24(8):2640. doi: 10.3390/s24082640 (PMC11053570; doi:10.3390/s24082640)
Supplement: Supplementary file 1 [file sensors-24-02640-s001.zip › sensors-2917004-supplementary.pdf]

Tables and Supplementary Figures

pilot (N variables) (N sessions)	model name	mape mean	mape std	pilot (N variables) (N sessions)	model name	mape mean	mape std
**P-1 (37)(42)**	**ab**	55.24	34.86	**P-10 (35) (60)**	**ab**	28.99	20.21
	**en**	43.17	25.08		**en***	23.06	6.12
	**gb**	58.54	35.48		**gb**	30.60	11.51
	**lstm**	66.74	68.51		**lstm**	58.97	20.40
	**pr**	43.73	25.13		**pr**	31.69	14.33
	**rf**	54.53	34.33		**rf**	31.91	14.09
	**sv**	49.57	28.58		**sv**	26.18	8.57
	**vr***	41.44	25.66		**vr**	27.67	14.39
**P-4 (29) (47)**	**ab**	24.46	28.92	**P-11 (34) (60)**	**ab**	26.29	8.81
	**en**	24.55	28.31		**en**	24.78	8.80
	**gb**	31.64	37.56		**gb**	26.61	8.06
	**lstm**	48.26	21.65		**lstm**	25.95	7.73
	**pr**	23.82	26.61		**pr**	25.53	9.92
	**rf**	31.52	36.19		**rf**	26.06	9.68
	**sv**	27.45	33.41		**sv**	26.84	11.11
	**vr***	22.87	28.54		**vr***	24.04	8.68
**P-5 (34) (56)**	**ab**	16.95	8.33	**P-14 (37) (60)**	**ab**	13.33	3.47
	**en**	16.54	6.43		**en**	13.01	5.65
	**gb**	17.44	5.13		**gb**	14.58	5.19
	**lstm**	19.51	5.72		**lstm**	14.04	4.46
	**pr**	16.60	6.23		**pr**	11.15	2.25
	**rf**	19.23	6.55		**rf**	13.64	4.75
	**sv**	17.01	5.51		**sv**	13.66	4.93
	**vr***	15.61	5.98		**vr***	9.03	3.32

**P-6 (36) (59)**	**ab***	22.73	6.10	**P-16 (30) (60)**	**ab**	11.33	1.93
	**en**	25.24	6.68		**en**	10.17	3.05
	**gb**	29.35	8.53		**gb**	11.60	3.94
	**lstm**	39.36	11.64		**lstm**	11.23	2.80
	**pr**	24.09	6.16		**pr**	11.08	2.93
	**rf**	24.56	7.23		**rf**	11.58	2.84
	**sv**	25.51	9.14		**sv**	10.87	2.30
	**vr**	24.19	8.67		**vr***	10.01	2.59
**P-7 (36) (45)**	**ab**	5.38	1.03	**P-20 (32) (60)**	**ab**	54.24	51.97
	**en**	5.79	1.18		**en**	52.49	43.18
	**gb**	5.92	2.11		**gb**	59.12	58.33
	**lstm**	13.67	6.21		**lstm**	91.11	76.43
	**pr**	6.00	1.61		**pr**	50.37	39.75
	**rf***	5.14	0.83		**rf**	57.90	59.76
	**sv**	6.04	1.08		**sv**	49.65	35.59
	**vr**	5.30	2.04		**vr***	48.10	33.62
**P-9 (32) (60)**	**ab**	16.20	6.71	**P-28 (27) (56)**	**ab**	63.84	51.79
	**en**	16.76	8.06		**en**	68.88	62.54
	**gb**	17.63	4.62		**gb**	63.17	48.06
	**lstm**	24.95	12.09		**lstm***	55.79	18.67
	**pr**	15.95	4.75		**pr**	63.18	47.52
	**rf**	15.85	5.90		**rf**	70.96	65.87
	**sv**	15.43	5.45		**sv**	76.24	59.00
	**vr***	14.44	5.45		**vr**	61.31	50.71

Supplementary Table S1: Individual wellbeing model performance for each subject across all models by MAPE (mean absolute percentage error) mean and standard deviation. Each subject name is followed by the number of independent features they had. Model names are as follows; ab: adaboost regressor, en: ensemble model; gb: gradient boost; lstm: long short term memory; pr:

poisson regressor; rf: random forrest; sv: support vector; vr: voting regressor. The best-fit model for each subject (i.e. with lowest MAPE) is denoted by *.

pilot (N variables)	model name	mape mean	mape std	pilot	model name	mape mean	mape std
**P-6 (36) (59)**	**ab**	20.27	10.57	**P-14 (37) (60)**	**ab**	11.43	4.02
	**en**	19.48	11.18		**en***	11.03	3.00
	**gb**	17.83	9.63		**gb**	11.09	4.34
	**lstm**	25.17	14.14		**lstm**	12.96	2.06
	**pr**	20.57	11.94		**pr**	11.21	3.39
	**rf**	19.98	9.92		**rf**	11.07	4.26
	**sv***	16.89	7.48		**sv**	11.54	1.81
	**vr**	17.33	10.05		**vr**	11.42	3.73
**P-7 (36) (45)**	**ab**	7.52	3.28	**P-16 (30) (60)**	**ab**	17.50	5.32
	**en***	7.22	2.81		**en**	17.21	4.73
	**gb**	9.60	3.48		**gb**	18.15	4.59
	**lstm**	13.73	7.88		**lstm**	17.15	3.95
	**pr**	7.22	2.81		**pr**	17.40	5.45
	**rf**	7.87	3.02		**rf**	17.90	5.22
	**sv**	7.22	2.81		**sv**	17.95	5.56
	**vr**	7.22	2.81		**vr***	16.36	3.92
**P-9(32) (60)**	**ab**	14.51	1.66	**P-20 (32) (60)**	**ab**	16.78	9.53
	**en**	14.09	3.77		**en**	17.06	8.17
	**gb**	14.93	1.86		**gb***	14.53	5.76
	**lstm**	18.24	2.13		**lstm**	23.22	10.86
	**pr**	14.77	3.78		**pr**	17.01	8.65
	**rf**	14.03	1.24		**rf**	14.78	5.04
	**sv**	14.70	6.76		**sv**	17.26	7.59
	**vr***	12.48	1.77		**vr**	15.03	7.19
					**ab**	18.08	4.98

**P-28 (27) (56)**	**en**	20.78	4.36
	**gb**	18.18	6.53
	**lstm**	18.86	4.60
	**pr**	17.96	2.36
	**rf**	23.55	5.00
	**sv**	27.41	7.29
	**vr***	16.89	4.03

Supplementary Table S2: Individual empathy model performance for each subject across all models by MAPE (mean absolute percentage error) mean and standard deviation. Each subject name is followed by the number of independent features they had. Model names are as follows, ab: adaptive boost regressor, en: ensemble model; gb: gradient boost; lstm: long short-term memory; pr: poisson regressor; rf: random forrest; sv: support vector; vr: voting regressor. The best-fit model for each subject (i.e. with lowest MAPE) is denoted by *.

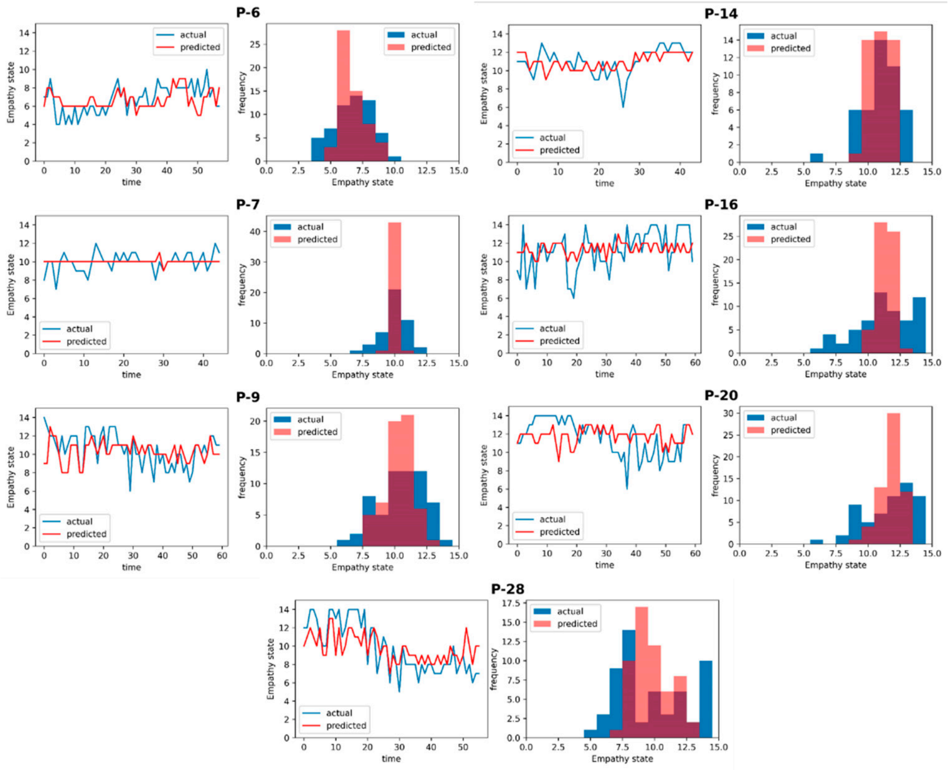

Supplementary Figure S1: Individual predictions and histograms for wellbeing. Line plots show the predictions across sessions.

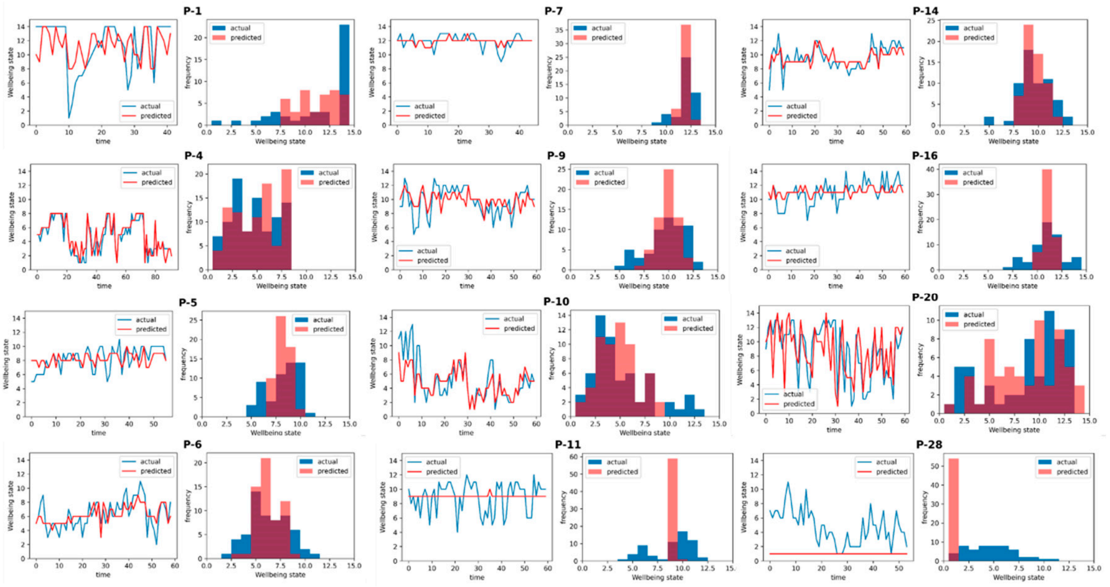

Supplementary Figure S2: Individual predictions and histograms for empathy. Line plots show the predictions across sessions.

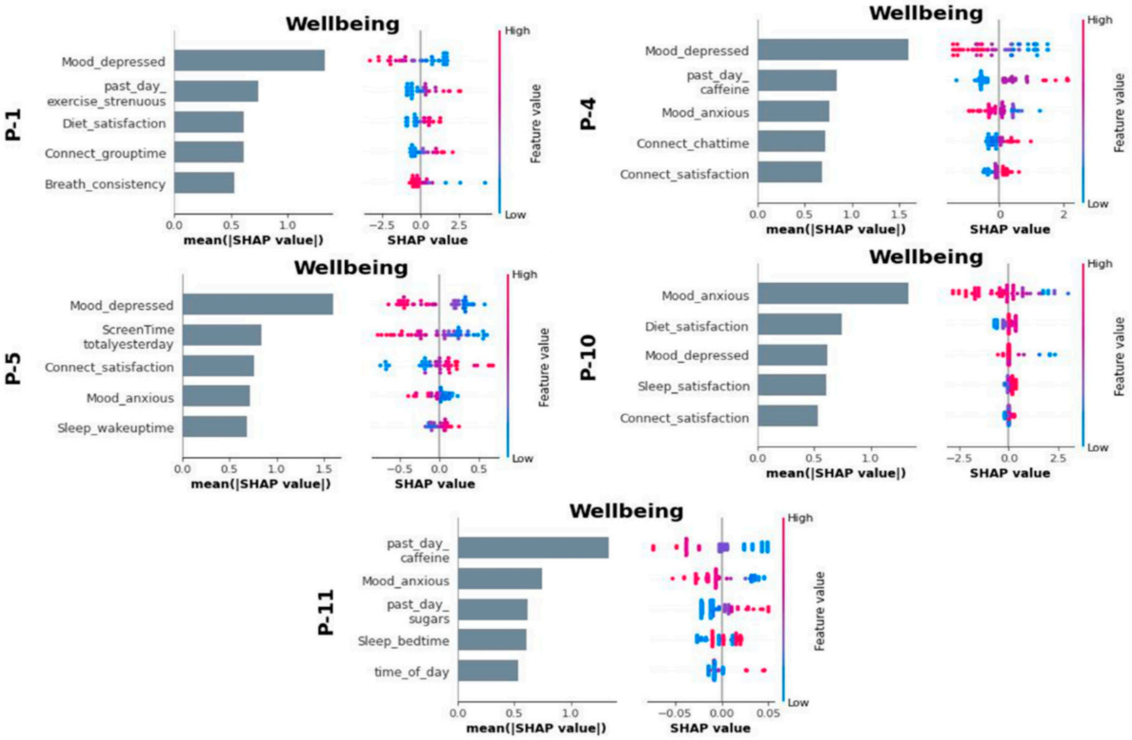

Supplementary Figure S3: SHapley Additive Explainer (SHAP) plots for subjects with only wellbeing model.
